# A 3D *in vitro* co-culture model to investigate tumor-endothelial interactions in Neurofibromatosis type 2-associated meningiomas

**DOI:** 10.1093/noajnl/vdag218

**Published:** 2026-08-17

**Authors:** Srirupa Bhattacharyya, Roberta L Beauchamp, Vijaya Ramesh

**Affiliations:** Department of Neurology, Center for Genomic Medicine, Massachusetts General Hospital, Boston, Massachusetts, USA; Harvard Medical School, Boston, Massachusetts, USA; Department of Neurology, Center for Genomic Medicine, Massachusetts General Hospital, Boston, Massachusetts, USA; Department of Neurology, Center for Genomic Medicine, Massachusetts General Hospital, Boston, Massachusetts, USA; Harvard Medical School, Boston, Massachusetts, USA

**Keywords:** 3D tumoroid model, Apelin (APLN), angiogenesis, mTORC1, NF2-associated meningioma

## Abstract

**Background:**

Neurofibromatosis type 2 (NF2)-associated meningiomas and schwannomas are vascular tumors, and while vascular endothelial growth factor (VEGF) inhibition with bevacizumab has benefited some NF2-related schwannomas, most NF2-associated meningiomas remain nonresponsive.

**Methods:**

Leveraging our transcriptomic data, we performed Gene Ontology (GO) analysis comparing NF2-deficient meningioma cells with NF2-expressing arachnoid cells (ACs). We then established a 3D *in vitro* angiogenesis model by co-culturing NF2-null meningioma cells with human umbilical vein endothelial cells (HUVECs). Endothelial sprouting was assessed by CD31/PECAM immunostaining. Effects of third-generation mechanistic target of rapamycin complex 1 (mTORC1)-selective inhibitor RMC-6272 as well as *APLN* knock-out using CRISPR-Cas9 gene editing were also examined.

**Results:**

GO analysis identified vascular development among the top significantly upregulated pathways in NF2-deficient cells. In 3D co-culture, ECs formed radially sprouting tube-like networks from the spheroid surface, and our data supports an angiogenesis phenotype driven by meningioma cells. Given these results along with hyperactivation of mTORC1 upon NF2*-*deficiency, we examined whether RMC-6272 disrupts meningioma-driven angiogenesis. RMC-6272 potently suppressed EC sprouting. Cross-referencing baseline transcriptomic data, we identified Apelin (*APLN*), the ligand for APLN receptor (APLNR), as a basally upregulated angiogenic factor in *NF2*-deficient meningiomas. Quantitative RT-PCR (qRT-PCR) confirmed increased *APLN* expression in *NF2*-null immortalized and patient-derived meningioma lines, with reduced expression upon mTORC1 inhibition. Apelin-13 stimulation enhanced sprouting, whereas *APLN* deletion reduced endothelial sprouting.

**Conclusions:**

Here we establish a 3D-tumoroid model and implicate tumor-derived Apelin as an important contributor to NF2-associated meningioma angiogenesis. Our data also suggest that *APLN* expression is regulated, at least in part, by mTORC1. Together, these results provide a preclinical platform for investigating angiogenic vulnerabilities beyond VEGF in *NF2*-deficient meningiomas.

Key Points

- *NF2*-deficient meningioma cells drive endothelial sprouting in a 3D tumor angiogenesis model.
- mTORC1 inhibition suppresses endothelial sprouting and network complexity.
- Apelin promotes sprouting, and *APLN* knockout in 3D tumoroids impairs endothelial outgrowth.

Importance of the Study

Neurofibromatosis type 2 (NF2)-associated meningiomas are often highly vascular and show limited responses to anti-vascular endothelial growth factor (VEGF) therapy, highlighting the need for improved treatment strategies. Here, we established a 3D meningioma-endothelial tumoroid model to evaluate tumor-driven angiogenesis in NF2-deficient meningioma. Using this model, we show that meningioma cells are sufficient to drive endothelial sprouting *in vitro* and that third-generation mechanistic target of rapamycin complex 1 (mTORC1)-selective inhibitor RMC-6272 markedly suppresses sprouting network formation. We further identify Apelin/*APLN* as a candidate effector associated with this angiogenic phenotype, showing that *APLN* is elevated in NF2-deficient meningioma lines, reduced following mTORC1 inhibition, promotes sprouting when added exogenously, and is required for efficient angiogenesis in meningioma tumoroids. Together, these findings support a role for tumor-derived Apelin in NF2-associated meningioma angiogenesis, establish a tractable preclinical platform for studying meningioma-endothelial interactions, and support a rationale to therapeutically target mTORC1-regulated angiogenic pathways.

NF2-related schwannomatosis (formerly known as neurofibromatosis type 2), hereafter referred as “NF2,” is a tumor-predisposition syndrome affecting approximately 1 in 33,000 individuals worldwide and characterized by the development of multiple nervous system neoplasms.^1^^,^^2^ Bilateral vestibular schwannomas are the hallmark of this condition, while meningiomas occur in approximately 80% of the patients.^3^ Although majority of tumors are histologically benign, compression of adjacent brain and spinal cord due to their location, their multiplicity, and frequent involvement of cranial nerves, spinal nerve roots, and peripheral nerves can result in substantial morbidity and, in many patients, premature mortality.^4^ In addition, meningiomas represent the most common primary intracranial tumor in adults.^5^ Somatic inactivation of *NF2* is also observed in approximately half of the sporadic meningiomas.^6^^,^^7^ While maximal surgical resection remains the standard of care,^5^ management is often limited by the anatomical location of these tumors and their highly vascular nature, which can increase intraoperative blood loss and perioperative risk.^8^ Thus, these tumors represent a persistent and significant unmet clinical need, affecting patients with the rare hereditary condition NF2, as well as the larger cohort with sporadic tumor presentations.

Given the pronounced vascularity of these tumors, bevacizumab, a vascular endothelial growth factor (VEGF) inhibitor, has been evaluated as an anti-angiogenic agent for NF2-associated tumors.^9^ However, despite its established benefit in progressive vestibular schwannomas, its efficacy in meningiomas has been limited, with only modest benefit reported.^10^^,^^11^ Furthermore, the weak correlation between VEGF expression and microvascular density in these tumors suggests that meningioma vascularization might be maintained through VEGF-independent mechanisms.^10^ While tumor cell-derived factors are known to contribute to vascularization, the specific mediators that engage endothelial cells and sustain this process in NF2-associated meningiomas remain poorly defined. These findings underscore the need to define alternative, potentially druggable pro-angiogenic pathways that operate downstream of, or in parallel with, canonical VEGF signaling.

NF2-associated tumors develop through loss of the tumor suppressor protein merlin.^12^ We and others have previously established that merlin functions as a negative regulator of mechanistic target of rapamycin complex 1 (mTORC1), and chronic mTORC1 activation is a hallmark of *NF2*-deficient tumors.^13^ As a central integrator of nutrient and growth signals, mTORC1 coordinates transcriptional and translational programs that influence vascular recruitment, including the regulation of Hypoxia-inducible factor 1-alpha (HIF-1α) and selective cap-dependent translation via the 4E-BP1/eIF4E axis.^14^ Although treatment with first-generation allosteric mTORC1 inhibitor rapamycin/rapalogs suppresses tumor angiogenesis in various contexts, clinical results with rapalogs in NF2 have been modest, likely due to incomplete suppression of the mTORC1-4E-BP1 axis.^15^^,^^16^ In this context, a next-generation bi-steric mTORC1-selective inhibitor, RMC-6272, more completely inhibits 4E-BP1 phosphorylation and demonstrates improved preclinical activity in *NF2*-deficient models.^17^

While prior studies have focused on the efficacy of mTORC1 inhibition in suppressing tumor growth,^18^ the specific contribution of this pathway to tumor-driven angiogenesis in *NF2*-deficient tumors is yet to be defined. Given its central role in regulating translation of selective mRNAs, mTORC1 is well positioned to control the production of pro-angiogenic factors within the tumor secretome^14^; however, directly linking mTORC1 activity to functional vascular outputs has been challenging in the context of NF2. Interrogating these processes requires model systems that capture paracrine tumor-endothelial interactions.^19^^,^^20^ Traditional 2D cultures lack the three-dimensional (3D) characteristics, such as hypoxic gradients and cell-cell interactions of the tumor microenvironment, while *in vivo* models show numerous pathophysiological and practical limitations.^21^ In this context, 3D vascularized tumoroid systems provide a tractable intermediate, preserving key physiologic features while enabling controlled perturbation of tumor and endothelial compartments and direct quantification of endothelial sprouting and vascular network formation.^22^

Here, we sought to define the role of mTORC1 in tumor-driven angiogenesis by establishing a biomimetic 3D meningioma-endothelial co-culture system that recapitulates key features of vascular morphogenesis *in vitro*. Using this platform, we delineated tumor-intrinsic contributions to neovascularization and assessed the impact of mTORC1 inhibition on endothelial sprouting and vascular network formation. We identify broad pro-angiogenic signatures potentially contributing to sustained meningioma vascularization independently of canonical VEGFA signaling. Within this framework, we highlight *APLN* as a candidate pro-angiogenic mediator whose expression is reduced following mTORC1 inhibition. Functionally, we show that exogenous Apelin-13 enhances endothelial sprouting, whereas CRISPR-Cas9-mediated deletion of *APLN* reduces sprouting in this assay. Collectively, these findings support a role for tumor-derived Apelin in NF2-associated meningioma angiogenesis and suggest that mTORC1-dependent regulation of *APLN* expression may represent a candidate therapeutic vulnerability.

## Methods

### Cell Lines and Reagents

Human cell lines used in this study included the *NF2-*deficient meningioma lines Ben-Men-1,^23^ MN1-LF,^24^ and KT21-MG,^25^ together with patient-derived lines MN597, MN617, MN619, MN621, MN626, MN644A, and MN647D, for which cell line establishment and growth conditions have been previously described.^26^ Surgical collection of tumors was performed following Massachusetts General Hospital IRB-approved Human Subjects protocols after informed consent, consistent. *NF2*-intact arachnoid cells (ACs) were used as controls. Meningioma cell lines were maintained in Dulbecco’s Modified Eagle Medium (DMEM) supplemented with 15% FBS and penicillin-streptomycin under standard humidified culture conditions at 37°C and 5% CO_2_. Human umbilical vein endothelial cells (HUVECs) were purchased from Lonza and maintained in EGM-2 endothelial growth medium (Lonza). RMC-6272 was generously provided by Revolution Medicines, and exogenous Apelin-13 was purchased from Phoenix Pharmaceuticals. Growth factor-reduced Matrigel (Sigma) was used for all hetero-spheroid embedding experiments. Alexa Fluor 647-conjugated CD31 antibody was obtained from Cell Signaling Technology, and the APLN receptor (APLNR) antibody was obtained from Bioss, and β-actin from Santa Cruz Biotechnology. Additional reagents, treatment concentrations, and treatment durations are described in the relevant figure legends.

### Gene Ontology Enrichment Analysis

Baseline transcriptomic analyses comparing *NF2*-intact ACs and *NF2*-deficient Ben-Men-1 cells have been previously described.^27^ To identify biological processes overrepresented in *NF2*-deficient meningioma cells, Gene Ontology (GO) enrichment analysis was performed using Enrichr^28-30^ on differentially expressed geneset with log_2_ fold change > 2 and Benjamini-Hochberg-adjusted *P* < .05. The top 10 overrepresented GO Biological Process terms were reported.

### cBioPortal Analysis

To evaluate the genomic features of *APLN* in meningioma, we analyzed a publicly available meningioma cohort from Nassiri et al^31^ (*n* = 121) using the cBioPortal for Cancer Genomics platform.^32^^,^^33^ The frequency of *APLN* genomic alterations, including somatic single-nucleotide variants (SNVs) and copy-number variations (CNVs), was assessed. In addition, we analyzed *APLN* mRNA expression in relation to available clinical features, including tumor recurrence status, WHO grade. Statistical analyses were performed to compare *APLN* expression between clinical groups, and a *P* value <.05 was considered statistically significant.

### Three-Dimensional Meningioma-Endothelial Tumoroid Generation

To model tumor-driven angiogenesis *in vitro*, we adapted and modified a previously described glioblastoma tumoroid protocol consisting of spheroid generation followed by extracellular matrix embedding.^34^ In the first step, meningioma-endothelial hetero-spheroids were generated using a scaffold-free forced-floatation approach in a 96-well format. Briefly, meningioma cells and endothelial cells were dissociated by trypsinization, and cell suspensions were centrifuged at 300 g for 5 min. Cell pellets were resuspended in serum-free Dulbecco’s Modified Eagle Medium (DMEM) for meningioma cells or EGM-2 for HUVECs. Meningioma and HUVEC cells were then combined at a 1:1 ratio, and 5,000 total cells/well were seeded into 96-well U-bottom microplates (Greiner) in a 1:1 mixture of EGM-2 and serum-free DMEM in a total volume of 200 μl. Plates were centrifuged at 1000 rpm for 10 min and incubated at 37°C and 5% CO_2_ for 48 h to form compact spheroids. Unless otherwise indicated, a 1:1 meningioma: HUVEC ratio was used for downstream all the experiments. A similar procedure was followed for AC-HUVEC heterospheroids. For HUVEC-only spheroids, 5,000 HUVEC cells/well were seeded in an equal mixture of DMEM and Endothelial Basal Medium (EBM).

Following spheroid maturation, defined as the period during which mixed meningioma and endothelial cells undergo self-assembly, aggregation, and compaction to form hetero-spheroids, 3-4 hetero-spheroids were transferred from the 96-well plate into Eppendorf tubes and allowed to settle. Medium was aspirated, 1 ml Phosphate-Buffered Saline (PBS) was added, and tubes were centrifuged at 300 g for 5 min. After aspiration of PBS, 100 μl of medium was added together with 100 μl growth factor-reduced Matrigel (Sigma) while samples were maintained on ice. For standard conditions, EGM-2 was used; for basal media conditions, EBM was used instead. All treatments and stimulations were added at this step prior to embedding.

For inhibitor studies, spheroids were treated with RMC-6272 or vehicle control. For ligand stimulation experiments, recombinant Apelin-13 was added at the indicated concentrations under reduced growth factor conditions. The resulting 200 μl suspension was transferred into wells of a μ-Slide 8-well high Grid-500 ibiTreat chamber slide (Ibidi) and incubated at 37°C and 5% CO_2_ for 24-72 h, as indicated in the respective experiments.

### Immunofluorescence Staining of the Tumoroids

To visualize endothelial sprouting, tumoroids were fixed in 4% paraformaldehyde (PFA) in PBS for 30 min at room temperature. Samples were then washed twice with PBS for 5 min each and incubated for 1 h at room temperature in permeabilization/blocking buffer containing 2% normal goat serum (NGS), 1% BSA, 0.5% Triton X-100, and 0.05% Tween-20 in PBS. After 3 PBS washes, tumoroids were incubated overnight at 4°C with anti-CD31/PECAM-1 (Cell Signaling Technology, Alexa Fluor 647-conjugated) diluted 1:100 in incubation buffer containing 2% NGS, 1% BSA, and 0.05% Tween-20 in PBS.

### Image Acquisition and Vascular Network Quantification

Z-stack images were acquired using a Yokogawa CQ1 system in brightfield and Alexa Fluor 647 channels. Images were captured at 6 μm intervals throughout the tumoroid stack. Maximum-intensity projection images were generated from the focal planes for each tumoroid. For quantification of angiogenic sprouts, the central spheroid core was masked using ImageJ. Endothelial networks were quantified using AngioTool version 0.6a,^35^ measuring vessel area, total vessel length, total number of junctions, and total number of endpoints. Vessel area was defined as the area occupied by segmented endothelial structures, total vessel length as the sum of all capillary-like structures, endpoints as open vessel termini, and junctions as branching points. Data were normalized to the indicated control condition and plotted using GraphPad Prism 10 software.

### CRISPR/Cas9-Mediated Disruption of *APLN*

To generate *APLN*-deficient meningioma cells, Ben-Men-1 cells were transfected with a commercial Apelin CRISPR/Cas9 Knockout plasmid pool (human; Santa Cruz Biotechnology, sc-401548) consisting of 3 plasmids, each encoding Cas9 nuclease and an *APLN*-specific 20-nt guide RNA. The guide sequences were CTCTCCTTGACCGCGGTGTG, AGACGGCAATGTCCGCCACC, and GGCCCATTCCTTGACCCTCT derived from the GeCKO v2 library and target the human *APLN* locus. Control plasmid encoding the Cas9 nuclease and a nonspecific 20 nt guide RNA was used as a control. Transfection was carried out following manufacturer’s guidelines. Briefly, 1.5 × 10^5^ cells were seeded into each well of a 6-well plate in 3 ml antibiotic-free standard growth medium and incubated for 24 h. Plasmid DNA was diluted in Plasmid Transfection Medium (Santa Cruz Biotechnology, sc-108062), and UltraCruz Transfection Reagent (Santa Cruz Biotechnology, sc-395739) was diluted separately in the same medium. Equal volumes were combined to form DNA-transfection reagent complexes and incubated for at least 20 min at room temperature before being added dropwise to cells in fresh antibiotic-free growth medium. Cells were then incubated under standard culture conditions for 24 h. Successful transfection was confirmed by GFP fluorescence. GFP-positive cells were then isolated by flow cytometric sorting, plated as single cells, and expanded clonally for downstream genotyping. Genomic DNA was isolated from expanded clones using a Qiagen genomic DNA isolation kit. The *APLN* region was amplified by PCR using primers flanking the CRISPR target site (F: 5′- GAGAGGAGGCTGCAGAAGAG-3′, R: 5′-R- GGGGCGCTCTGTCAATCTG-3′), and PCR products were analyzed by Sanger sequencing. Chromatograms were aligned to the wild-type *APLN* sequence to identify insertion/deletion events. Clones were classified as wild-type or null based on sequence analysis.

### RNA Extraction and Quantitative RT-PCR

Total RNA was extracted using TRIzol reagent (Thermo Fisher) according to the manufacturer’s instructions, followed by chloroform phase separation and purification on Qiagen RNeasy columns (Qiagen). cDNA synthesis was performed using the SuperScript VILO cDNA synthesis kit (Life Technologies). Quantitative RT-PCR (qRT-PCR) was performed in 3 biological replicates, each in triplicate, on a Roche LightCycler 480 (software version 1.5.0 SP3) using LightCycler 480 SYBR Green I Master Mix (Roche). Human *APLN* primers included APLN-F: 5′-GTCTCCTCCATAGATTGGTCTGC-3′ and APLN-R: 5′-GGAATCATCCAAACTACAGCCAG-3′ (PrimerBank ID 21314668a1). The internal control used was human *GAPDH* (hGAPDH-F: 5′-CCCCGGTTTCTATAAATTGAGC-3′ and hGAPDH-R: 5′-CACCTTCCCCATGGTGTCT-3′). Melt-curve analysis demonstrated single-peak specificity for each primer set. Relative expression was calculated using the comparative Ct method, and values were normalized to control samples, which were set to 1.0. Data are presented as dot plots as mean ± SEM.

### Immunoblotting

For Ben-Men-1, MN1-LF, KT21-MG1, and primary MN lines, cell lysis, SDS-polyacrylamide gel electrophoresis (PAGE), and immunoblotting were carried out as previously described^13^ with visualization of protein bands by standard chemiluminescence methods using either Amersham hyperfilms (Cytiva), an iBright FL1500 (Invitrogen) imaging system.

### Immunofluorescence Staining of Human Meningioma Tissue

To assess the spatial relationship between APLNR expression and tumor vasculature, immunofluorescence staining was performed on formalin-fixed, paraffin-embedded (FFPE) sections from a grade I meningioma specimen. Tissue sections were cut at 8 μm and mounted on microscope slides, then baked for 45 min at 60°C. For deparaffinization and rehydration, slides were washed twice in xylene for 3 min each, followed by xylene : 100% ethanol (1:1) for 3 min, 2 washes in 100% ethanol for 3 min each, 2 washes in 95% ethanol for 3 min each, then sequential washes in 70% ethanol and 50% ethanol for 3 min each, followed by rinsing in running distilled water for 5 min. Antigen retrieval was performed by boiling slides in 0.01 M sodium citrate buffer (pH 6.0) at 100°C for 20 min, followed by cooling at room temperature in the same buffer for 20 min. Slides were rinsed twice with 1× Tris-buffered saline containing 0.1% Tween-20 (TBST) for 5 min each, blocked in 5% serum or BSA for 2 h at room temperature, and incubated overnight at 4°C with primary antibodies against APLNR (Bioss, bs-21310R) and CD31/PECAM diluted in blocking buffer. After two washes in TBST, slides were mounted using ProLong Gold Antifade Mountant (Invitrogen). Images were acquired using Nikon AX1R confocal microscope.

### Statistical Analysis

Data are presented as mean ± SEM. For qRT-PCR, experiments were performed in 3 biological replicates, each in technical triplicate. For tumoroid angiogenesis assays, experiments were performed in 3 biological replicates, with each biological replicate containing a minimum of 2 tumoroids per condition. Comparisons between 2 groups were performed using Student’s *t* test. A value of *P* < .05 was considered statistically significant.

## Results

### *NF2*-Deficient Meningiomas Demonstrate Increased Endothelial Sprouting in 3D Tumoroids

**Figure 1.**
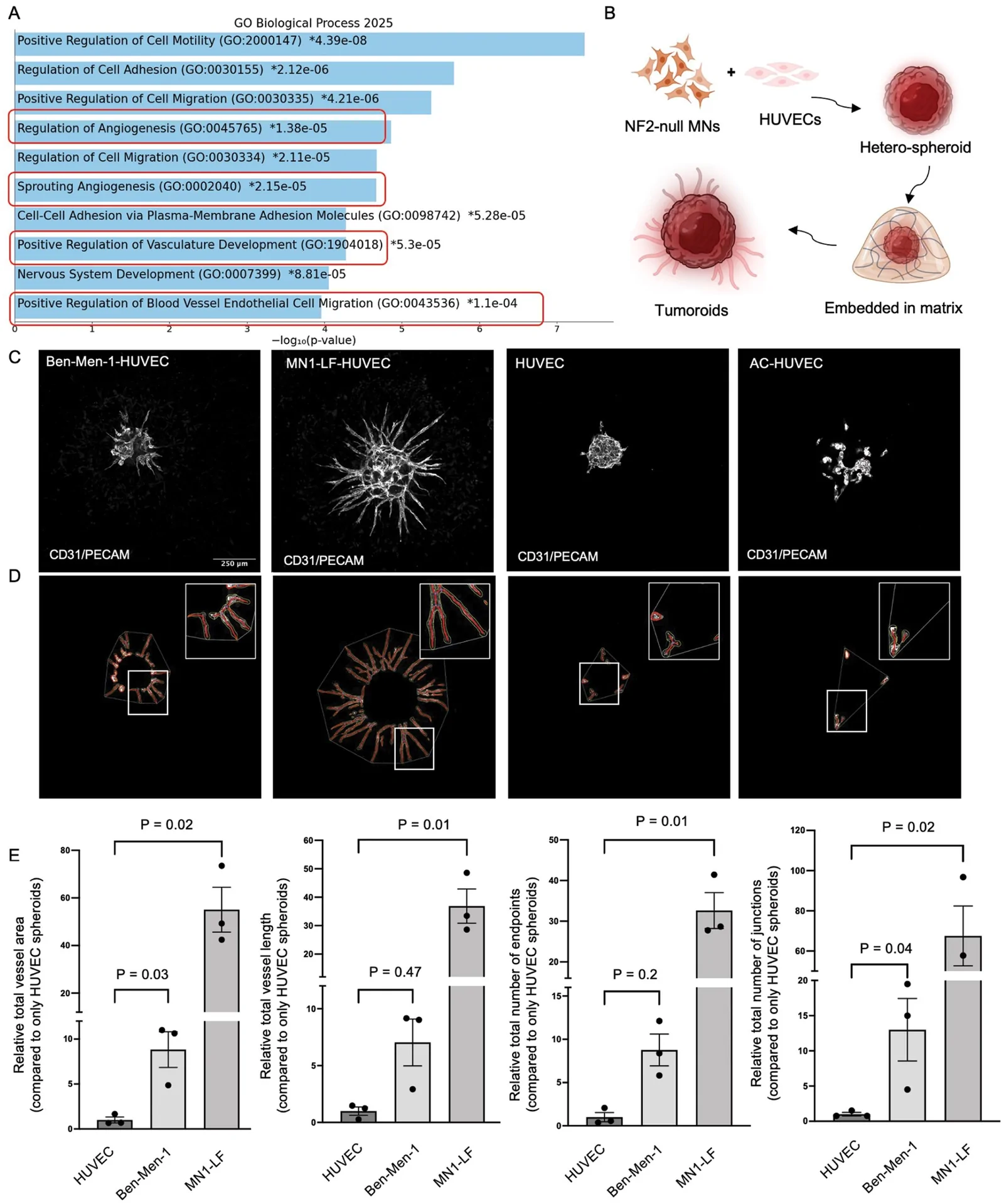
*NF2*-deficient meningioma models show a pro-angiogenic transcriptomic signature and drive endothelial sprouting in 3D tumoroid model. (A) Bar chart of top enriched terms from the GO Biological Process 2025 gene set library. The top 10 enriched terms for the input gene set are displayed based on the -log10 (*P*-value), with the actual *P*-value shown next to each term. (B) Schematic representation of the 3D hetero-spheroid angiogenesis assay. *NF2*-null meningioma (MN) cells were co-cultured with human umbilical vein endothelial cells (HUVECs) to generate hetero-spheroids, which were subsequently embedded in extracellular matrix followed by fixing and staining with CD31/PECAM to assess endothelial sprouting. (C) Representative maximum intensity projection images of CD31/PECAM positive hetero-spheroids generated with the *NF2-*deficient meningioma cell lines Ben-Men-1 and MN1-LF along with AC-HUVEC and HUVEC-only control spheroids. *NF2*-deficient meningioma-HUVEC tumoroids exhibited marked radial endothelial outgrowth, whereas HUVEC-only and AC-HUVEC spheroids showed limited sprouting (Scale bar denotes 250 µM). (D) Corresponding skeletonized images generated from the CD31 signal using AngioTool following spheroid core masking using ImageJ/Fiji. (E) Quantification of vascular parameters demonstrated that *NF2*-deficient meningioma cells significantly enhance endothelial network formation relative to control conditions. Bar graphs represent relative total vessel area, length, number of end points, and junctions across all the groups. All values are normalized to HUVEC-only spheroid control. *NF2*-deficient meningioma lines (Ben-Men-1 and MN1-LF) show a significant increase in all vascular metrics. Bar graphs were generated using GraphPad Prism 10. Data are presented as mean ± SEM from *n* = 3 biological replicates with at least *n* = 2 tumoroids per condition. Each dot represents the mean of the technical replicates within a single biological replicate. *P*-values have been calculated using a one-tailed Student’s *t*-test.

Leveraging our earlier transcriptome and drug screening studies in *NF2*-null meningioma cellular models,^27^ here we performed Gene Ontology (GO) enrichment analysis on transcripts significantly upregulated in *NF2*-deficient Ben-Men-1 cells (log_2_ fold change > 2) when compared with *NF2*-sufficient human arachnoidal cells (ACs). GO analysis revealed pathways associated with vascular regulation, including “regulation of angiogenesis,” “sprouting angiogenesis,” “positive regulation of vascular development,” and “positive regulation of blood vessel endothelial cell migration,” to be among the top 10 significantly enriched in Ben-Men-1 cells (Figure 1A). In addition, an *a priori* search of our transcriptome data identified several genes reported to play a role in angiogenesis that were significantly upregulated (log2 fold change > 2) in *NF2*-deficient meningioma cells (Table 1). Notably, canonical *VEGFA* was not significantly upregulated, supporting the notion of alternative pathways underpinning the enhanced vascular phenotype of *NF2*-deficient meningiomas.

**Table 1.** Angiogenic factor genes showing increased expression in Ben-Men-1 cells, *P*-value is BH adjusted; log2FC, log2 fold change

Gene ID	Protein name	log2FC	*P*-value
*SFRP1*	Secreted frizzled related protein 1	9.59	1.44E-19
*SEMA4D*	Semaphorin 4 D	7.93	1.84E-10
*FGF13*	Fibroblast growth factor 13	7.62	8.74E-07
*HBEGF*	Heparin-Binding EGF-like Growth Factor	6.76	5.2E-15
*ANXA3*	Annexin A3	6.11	5.13E-13
*APLN*	Apelin	5.88	7.26E-13
*IGF2*	Insulin-like growth factor 2	5.23	3.2E-09
*ANGPTL4*	Angiopoietin like 4	5.20	2.25E-07
*ENG*	Endoglin	3.52	1.16E-11
*DLL1*	Delta-like canonical Notch ligand 1	3.38	2.22E-06
*FGF1*	Fibroblast growth factor 1	2.47	0.00000219
*VEGFC*	Vascular endothelial growth factor C	2.21	1.80E-05
*THBS1*	Thrombospondin 1	2.11	4.01E-08

As an initial step to functionally examine potential mechanisms of vascularization in *NF2*-deficient meningiomas, we first established an *in vitro* biomimetic 3D “tumoroid” co-culture system to assess meningioma-driven effects on endothelial sprouting in a physiologically relevant microenvironment. We implemented a two-step protocol: (i) hetero-spheroids were generated using a scaffold-free forced-flotation method by co-culturing *NF2*-deficient meningioma cells with HUVECs in a 96-well format, producing uniform single spheroids, followed by (ii) embedding of hetero-spheroids, after 48 h of maturation, in growth factor-reduced Matrigel (diluted 1:1 in endothelial cell growth medium) and incubation for 72 h to allow formation of tumoroids (Figure 1B). Endothelial sprouting in the tumoroids was assessed by immunofluorescent staining with endothelial marker CD31/PECAM (Figure 1B, schematic). During optimization, various HUVEC: meningioma ratios were evaluated (4:1, 3:1, 2:1, and 1:1), and we observed that induction and maturation of vessel-like structures were dependent on the stoichiometric balance between tumor and endothelial cell populations. We found that a 1:1 ratio of HUVEC: meningioma cells yielded the most reproducible and well-organized networks, with higher proportions of meningioma cells driving enhanced sprouting angiogenesis (data not shown). HUVECs co-cultured with *NF2*-deficient meningioma lines (Ben-Men-1 or MN1-LF) exhibited robust, radially oriented CD31+ capillary-like outgrowths extending from the spheroid surface into the surrounding matrix, exhibiting sprouting morphologies reminiscent of nascent vessel formation. In contrast, spheroids generated from HUVECs co-cultured with ACs or HUVEC alone failed to form organized sprouting networks under identical conditions, indicating that angiogenic behavior is conferred specifically by the meningioma cells (Figure 1C). Although the endothelial cells in HUVEC-only spheroids organized into capillary-like networks within the spheroid body, they did not produce extensive radial sprouting into the surrounding matrix, supporting the concept of a meningioma cell-specific contribution toward neo-angiogenesis. Vascular network quantification was then performed using a computational pipeline with ImageJ/Fiji and AngioTool,^35^ and all metrics were normalized to HUVEC-only spheroids to define tumor cell-specific contributions to sprouting (Figure 1D and E). Although MN1-LF tumoroids appeared to generate more extensive sprouting than Ben-Men-1 tumoroids, both models demonstrated significantly increased sprouting angiogenesis compared with HUVEC-only controls. Additionally, we carried out embedding and tumoroid formation assays in basal medium lacking exogenous angiogenic factor supplements, which revealed similar capacity for endothelial sprouting, confirming that factors contributing to enhanced sprouting are indeed originating from meningioma tumor cells (Supplementary Figure S1). Collectively, these data validate the functional relevance of the transcriptomic pro-angiogenic signature and establish a 3D “tumoroid” platform suitable for mechanistic interrogation of meningioma-driven neovascularization as well as preclinical evaluation of anti-angiogenic therapies. Moreover, we also demonstrate that tumor-derived factors are sufficient to sustain the angiogenic program in the absence of exogenous cues.

### mTORC1 Inhibition Impairs Endothelial Sprouting in NF2-Deficient Tumoroids

**Figure 2.**
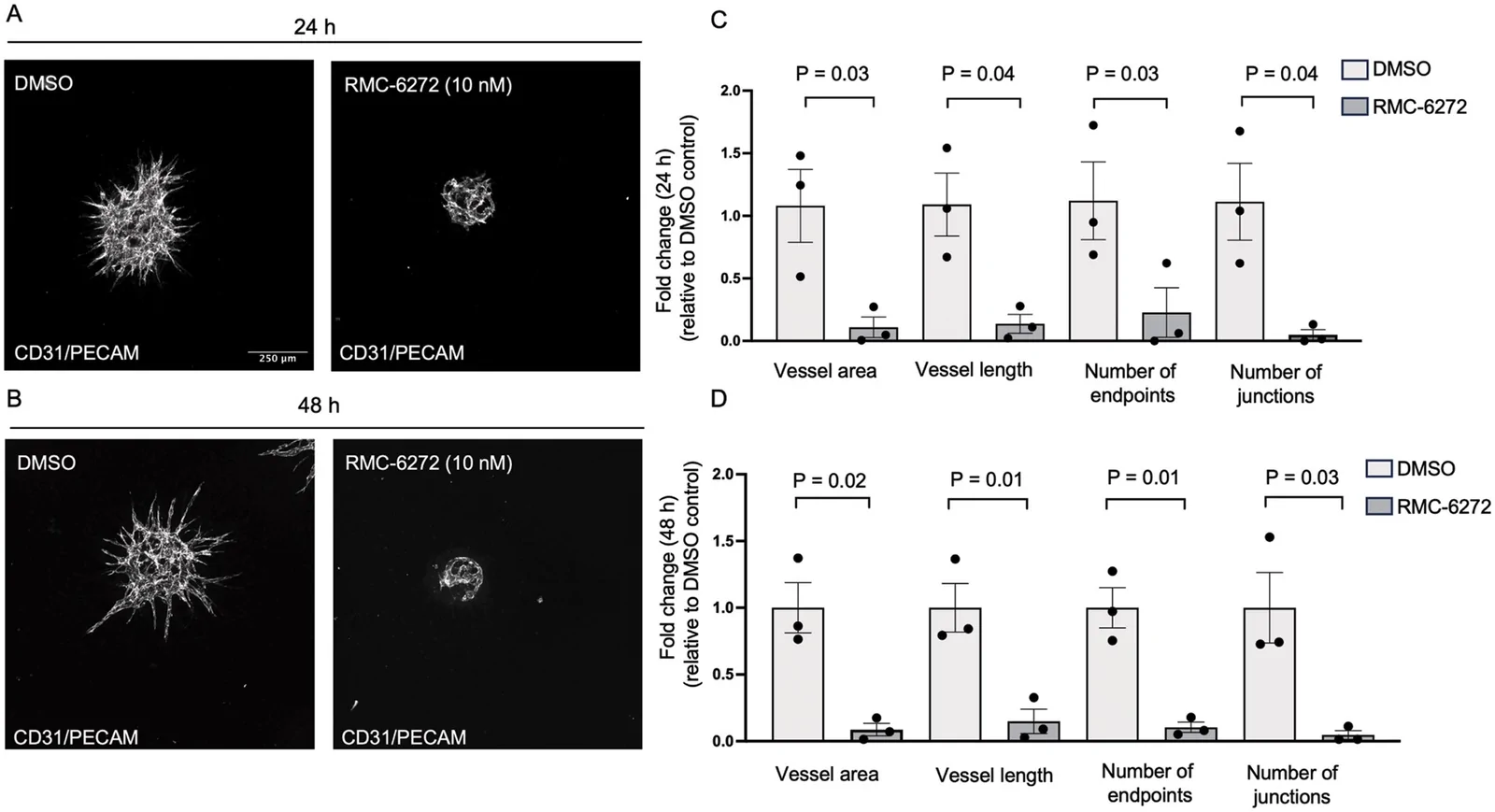
mTORC1 inhibition with RMC-6272 suppresses angiogenic sprouting in meningioma tumoroids. (A, B) Representative maximum intensity projection images of CD31/PECAM positive *NF2*-deficient meningioma tumoroids treated with DMSO or RMC-6272 (10 nM) and analyzed at 24 h (A) and 48 h (B). DMSO-treated tumoroids exhibited robust radial endothelial sprouting, whereas RMC-6272-treated tumoroids showed suppression of endothelial outgrowth. Scale bar = 250 μm (C-D) Quantitative analysis of vascular sprouting angiogenesis parameters at (C) 24 hours and (D) 48 hours. Metrics include vessel area, length, endpoints, and junctions. Treatment with 10 nM RMC-6272 resulted in a significant reduction in all the vascular metrics at both time points compared to DMSO controls. Data are normalized to time-matched DMSO controls and presented as mean ± SEM from *n* = 3 biological replicates with a minimum of *n* = 2 tumoroids per condition. Each dot represents the mean of the technical replicates within a single biological replicate. Bar graphs have been generated using GraphPad Prism 10. *P*-values were calculated using one-tailed Student’s *t* test.

Given our previous work establishing constitutive mTORC1 activation downstream of *NF2* loss,^13^ as well as documented links between mTORC1 signaling and tumor neo-vascularization,^14^^,^^36-38^ we next tested whether sprouting in *NF2*-deficient meningioma tumoroids is sensitive to mTORC1 inhibition. First, hetero-spheroids were generated as outlined above by co-culturing *NF2*-deficient meningioma cells (MN1-LF) with HUVECs (1:1). Next, the hetero-spheroids were embedded in Matrigel in the presence of 10 nM RMC-6272, based on prior studies in which 10 nM RMC-6272 treatment led to robust suppression of 4E-BP1 phosphorylation and reduction in global protein synthesis, without significant induction of apoptosis or cell death in *NF2*-deficient meningioma models.^17^ Hetero-spheroids embedded in Matrigel containing 0.1% Dimethyl Sulfoxide (DMSO) served as a control. Embedded hetero-spheroids were incubated for 24 and 48 hours, after which samples were fixed and immunostained for the endothelial marker CD31 to assess sprout formation. We observed radially oriented CD31-positive capillary-like sprouts invading the surrounding matrix at both time points in DMSO-treated controls. In contrast, RMC-6272 treatment elicited a robust reduction in nascent blood vessel-like structures (Figure 2A and B, representative images). Quantitative analyses at 24 h and 48 h, using Angiotool, with all parameters normalized to DMSO-control, revealed a significant reduction of (i) reduced total sprouting area (9.9-fold in 24 h and 11.5-fold in 48 h) indicative of restricted radial expansion of CD31-positive endothelial structures, (ii) total vessel length (7.9 -fold in 24 h; 6.7-fold in 48 h) consistent with diminished cumulative sprout elongation as (iii) a decrease in the total number of endpoints (4.9-fold in 24 h; 9.6-fold in 48 h) as well as (iv) a reduction in total junctions (22.6 -fold in 24 h and 38.8-fold in 48 h) reflecting a marked loss of branching complexity and network connectivity (Figure 2C and D). Taken together, these findings demonstrate that mTORC1 inhibition by RMC-6272 at 10 nM disrupts endothelial sprouting, branching, and radial vascular expansion in 3D *NF2*-deficient meningioma tumoroids consistent with sustained impairment of tumor-associated vascular network formation and organization.

### mTORC1 Inhibition Downregulates Apelin Expression

Given our data showing significant reduction in angiogenic sprouting upon mTORC1-inhibition, we next sought to identify potential candidate downstream mediators. Although mTORC1 is classically linked to tumor angiogenesis through HIF-1α/VEGF-associated pathways,^14^ baseline transcriptomic profiling of our *NF2*-deficient meningioma cell models did not reveal significant upregulation of *VEGFA*, suggesting the potential role of alternative mTORC1-regulated ligands.^27^ Among the shortlisted candidates, *APLN* emerged as a plausible effector. Prior studies have implicated Apelin/APLNR signaling in embryonic and tumor angiogenesis,^39^ endothelial tip-cell behavior and sprouting,^40^ vascular repair and maintenance,^41^ and adaptive responses to anti-angiogenic therapy upon VEGF-pathway inhibition.^42^^,^^43^ To determine whether *APLN* expression was associated with clinical outcomes and tumor grade, we analyzed a publicly available meningioma cohort^31^ using cBioPortal.^32^ In this cohort (*n* = 121), *APLN* expression was significantly higher in recurrent tumors compared with non-recurrent tumors (median expression: 1.243 vs. 0.34; *P* = .019) (Supplementary Figure S2A). Next, to assess whether increased *APLN* expression in meningioma is associated with genomic alterations, we analyzed matched whole-exome sequencing data from this cohort, and no somatic SNVs or copy-number alterations (CNVs) were identified, suggesting that *APLN* upregulation is unlikely to result from recurrent genomic alterations of the *APLN* locus. In addition, we found that *APLN* expression increased progressively across WHO grades 1, 2, and 3 in the meningioma cohort (median expression: 0.73, 1.16, and 1.24, respectively) although this trend did not reach statistical significance (Supplementary Figure S2B). Together with prior evidence where Apelin has been linked to tumor angiogenesis or vascular remodeling in multiple tumor types,^44-46^ these findings support the possibility that *APLN* may similarly sustain vascularization in *NF2*-deficient meningiomas.

**Figure 3.**
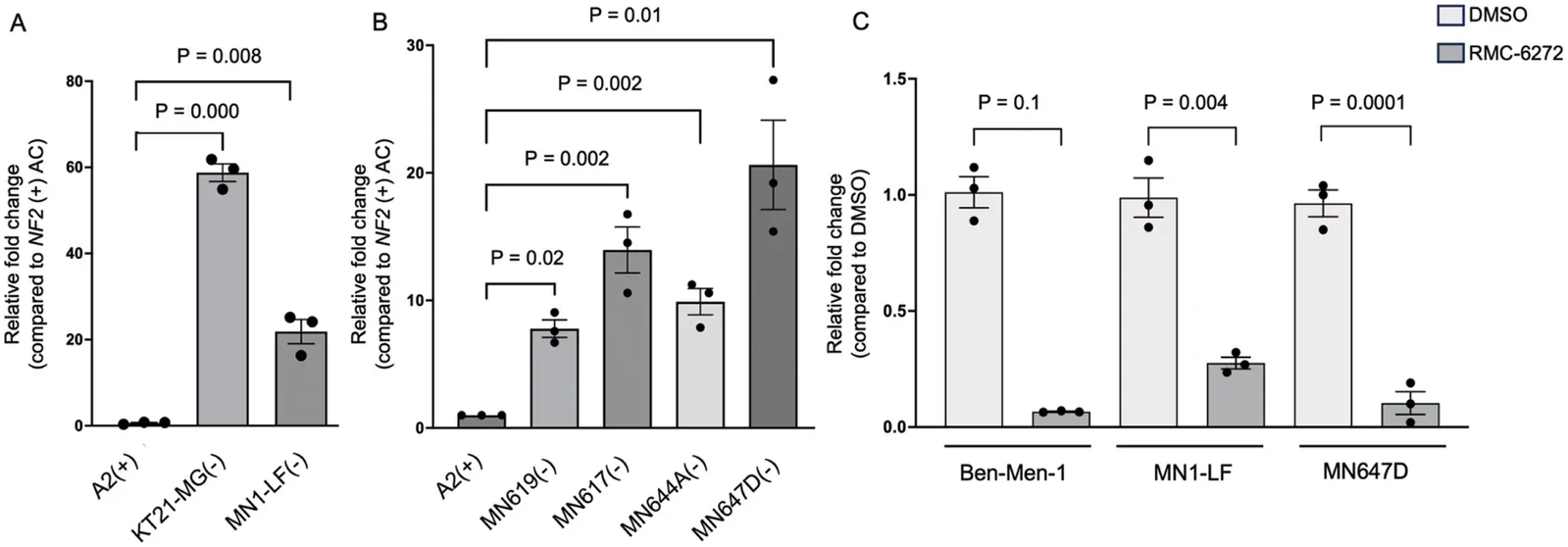
*APLN* is upregulated in *NF2*-deficient meningioma cells and is reduced by mTORC1 inhibition. (A-B) Quantitative RT-PCR analysis of *APLN* mRNA levels in (A) *NF2*-deficient immortalized meningioma cell lines (KT21-MG and MN1-LF) and (B) patient-derived *NF2*-deficient meningioma lines (MN619, MN617, MN644A, and MN647D) showing significant increase in *NF2*-deficient cells relative to *NF2*-intact control arachnoid cells (ACs). All data have been normalized to *GADPH*. (C) Relative fold change of *APLN* mRNA in *NF2*-deficient cells following treatment with the third-generation mTOR inhibitor RMC-6272, demonstrating significant reduction in *APLN* levels upon treatment. Bar graphs have been generated using GraphPad Prism 10. Data are normalized to DMSO controls and are represented as mean ± SEM.

As we previously reported, *APLN* expression is significantly increased in *NF2*-null meningioma line Ben-Men-1 relative to *NF2-*wild-type AC controls, the cell type of origin for meningioma.^24^ Extending our analyses to additional *NF2*-null meningioma lines, quantitative RT-PCR of *APLN* revealed increases of 60-fold in KT21-MG, 20-fold in MN1-LF, and increases in primary MN lines (MN617, MN619, MN644A, and MN647D) ranging from 8- to >20-fold (Figure 3A and B). Further, to determine whether *APLN* expression is affected by mTORC1 inhibition, Ben-Men-1, MN1-LF, and MN647D cells were treated with RMC-6272 (10 nM) for 24 h. Relative to DMSO controls, RMC-6272 treatment significantly reduced *APLN* transcript levels by approximately 70%-90% in all the cell lines, indicating that *APLN* expression is, at least in part, mTORC1-dependent (Figure 3C). Additionally, immunoblotting of *NF2*-null meningioma lines showed robust expression of APLN receptor (APLNR), and fluorescent immunohistochemical staining of a primary meningioma tumor (grade I) from a NF2 patient demonstrated APLNR positivity throughout as well as areas of co-staining with the endothelial marker CD31/PECAM. Notably, APLNR appeared to localize along and adjacent to vascular structures, with regions of partial overlap at the tumor-endothelial interface (Supplementary Figures S3 and S4), suggesting relevance of Apelin/APLNR in these tumors. Overall, these findings support a model in which mTORC1 inhibition attenuates tumor-driven angiogenesis, at least in part, through suppression of Apelin expression.

### Exogenous Apelin Augments Sprouting Under Growth Factor-Restricted Conditions

**Figure 4.**
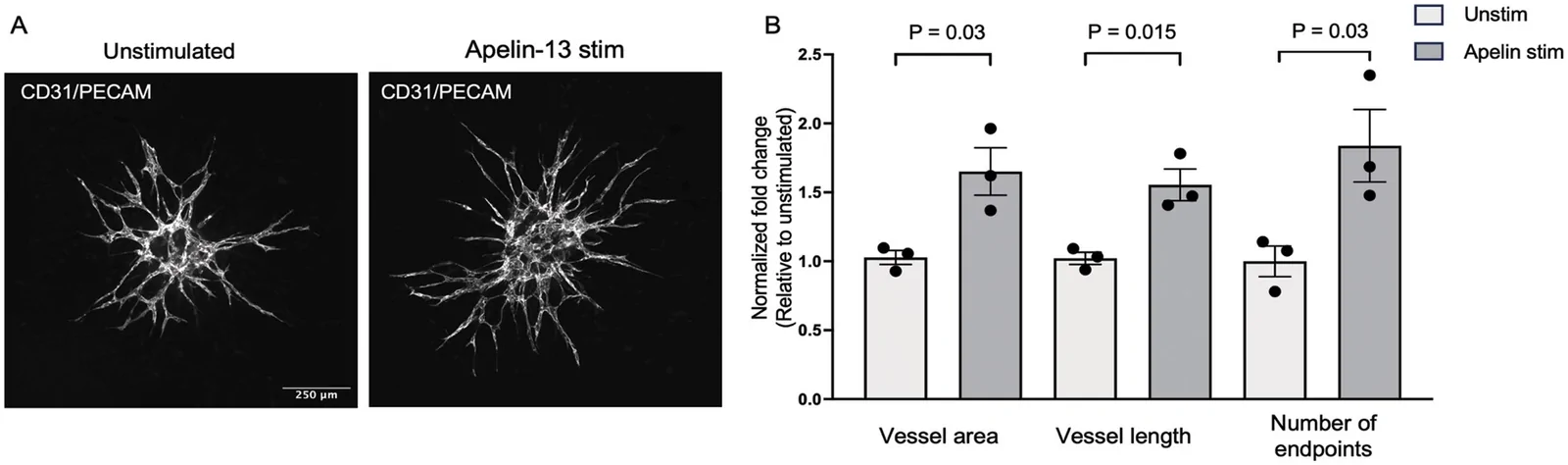
Apelin stimulation promotes angiogenesis in the meningioma tumoroids. (A) Representative maximum intensity projection images of hetero-spheroids subjected to exogenous Apelin-13 stimulation (unstimulated control, 5 µM apelin stimulation). Scale bar, 250 μm. (B) Quantification of vascular morphological parameters showing the effect of Apelin-13 on key angiogenic metrics: vessel area, vessel length, and number of end points. All data are normalized to unstimulated control. Data are presented as mean ± SEM from *n* = 3 biological replicates with at least *n* = 2 tumoroids per condition. *P*-values were calculated using one-tailed Student’s *t* test.

We next examined whether Apelin stimulation is sufficient to promote endothelial sprouting in our *NF2*-null meningioma tumoroid model. To test this, meningioma-endothelial hetero-spheroids were embedded in growth factor-reduced Matrigel mixed 1:1 with basal medium, in the presence or absence of Apelin-13, and endothelial sprouting was assessed by CD31 immunofluorescence. Apelin stimulation (5 μM) increased endothelial outgrowth from *NF2*-deficient tumoroids relative to unstimulated controls (Figure 4A). Quantitative analysis showed that Apelin significantly increased total vessel area (1.56-fold) and total vessel length (1.5-fold). In addition, the total number of endpoints was significantly elevated (1.8-fold), consistent with enhanced sprouting and branching activity (Figure 4B). Although the total number of junctions did not reach statistical significance, it showed an increasing trend (1.8-fold) in Apelin-treated tumoroids compared to unstimulated controls (Supplementary Figure S5). Together, these findings demonstrate that Apelin is sufficient to enhance endothelial sprouting in *NF2*-deficient meningioma tumoroids.

### Knockout of *APLN* Reveals Genotype-Dependent Angiogenic Phenotype of the Tumoroids

**Figure 5.**
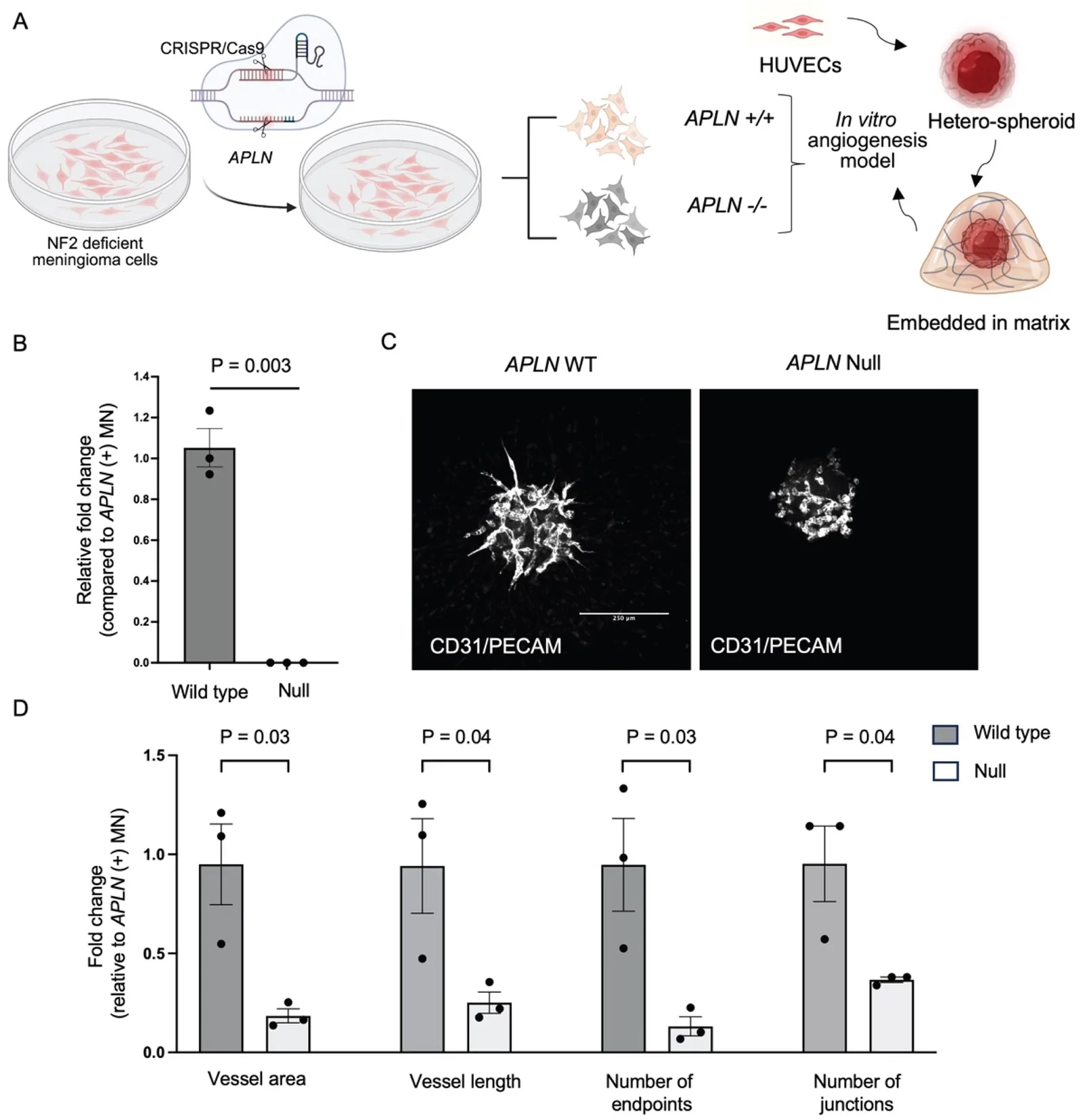
CRISPR/Cas9-mediated deletion of *APLN* impairs tumor-driven endothelial sprouting. (A) Schematic of the workflow used to generate *APLN*-deficient meningioma cells and assess their angiogenic potential in the 3D vascularized tumoroid model. (B) qRT-PCR validation of *APLN* expression in CRISPR-edited Ben-Men-1 clones showing relative *APLN* mRNA levels for homozygous null clone, normalized to the wild-type control. (C) Representative CD31/PECAM immunofluorescence images of endothelial structures in 3D vascularized tumoroids generated using *APLN* wild-type or *APLN*-null meningioma cells. Scale bar, 250 μm. (D) Quantification of vascular parameters in tumoroids generated with *APLN* wild-type and *APLN*-null meningioma cells. Bar graphs demonstrate significant reductions in total vessel area, total vessel length, total number of endpoints, and total number of junctions, indicating impaired endothelial sprouting, branching, and overall vascular network organization in the absence of tumor cell-derived Apelin. Data are presented as mean ± SEM from *n* = 3 biological replicates with a minimum of *n* = 2 tumoroids per replicate. Statistical significance was determined using one-tailed Student’s *t-*test.

To define the contribution of basal *APLN* upregulation in meningioma neo-angiogenesis, we next sought to inhibit the Apelin pathway in our tumor-endothelial co-culture system. We initially evaluated available pharmacologic APJ antagonists, including ML221 and MM54, but did not observe a suppression of endothelial sprouting consistent with prior reports indicating the limitations of the currently available APJ antagonists.^47^ We therefore chose to carry out CRISPR/Cas9 genome editing to directly knock out Apelin in Ben-Men-1 meningioma cells and then used *APLN*-null (−/−) or *APLN*-expressing (+/+) Ben-Men-1 to generate our tumoroid model (Figure 5A). Genotypic analysis identified 2 independent clones harboring a homozygous “T” insertion in exon 1 of *APLN* as well as several independent *APLN*-wild-type (WT) clones (Supplementary Figure S6). The insertion event was predicted to induce a frameshift mutation resulting in premature stop codons, consistent with loss-of-function. We next selected a *APLN*-null clone and a matched WT clone, for downstream studies. We first confirmed loss of *APLN* expression in the null clone compared to a WT clone by qRT-PCR (Figure 5B). We then used this pair to assess the effect of Apelin loss on tumor-driven angiogenesis using the 3D vascularized tumoroid platform. *APLN-*WT and *APLN*-null meningioma cells were co-cultured with HUVECs, embedded in extracellular matrix, and angiogenic sprouting was evaluated at 72 h. WT tumoroids formed radially organized capillary-like networks, whereas *APLN-*null tumoroids display a marked reduction in organized sprouting structures (Figure 5C). Quantitative analysis revealed significant decreases across multiple vascular parameters in the *APLN-*null tumoroids. Total vessel area was significantly decreased by 5.5-fold in *APLN-*null relative to WT, while total vessel length was reduced by 4.4-fold. In addition, total number of endpoints, reflecting active sprouting, was reduced by 7.1-fold, along with a 2.7-fold decrease in total number of junctions. Taken together, our results demonstrate that loss of *APLN* significantly impairs endothelial sprouting and network formation in *NF2*-deficient meningioma tumoroids, supporting Apelin as a functional contributor to the tumor-driven angiogenic phenotype for these tumors.

## Discussion

The clinical management of NF2-associated meningiomas remains challenging, particularly given the limited efficacy of VEGF-inhibitor bevacizumab in these tumors.^10^^,^^11^ Leveraging our previous transcriptomic analysis of *NF2*-deficient meningioma cells, we identified several pro-angiogenic factors, including *APLN, HBEGF, VEGFC, and SEMA4D,* to be markedly upregulated (Table 1). These factors engage diverse angiogenic signaling pathways, including VEGF/VEGFR (*VEGFC*), FGF/FGFR (*FGF1* and *FGF13*), EGFR (*HBEGF*), IGF (*IGF2)*, Notch (*DLL1*), TGF-β/endoglin (*ENG*), Apelin/APLNR (*APLN*), and semaphorin (*SEMA4D*) signaling. Many of these pathways converge on shared endothelial processes, including proliferation, migration, sprouting, and vascular remodeling. Collectively, these findings support the presence of a broad pro-angiogenic network independent of *VEGFA*, underscoring the complexity of the tumor-endothelial interface in *NF2*-deficient meningiomas. To investigate the mechanisms that sustain tumor-associated vascularization, we have established a novel biomimetic 3D co-culture tumoroid platform that models sprouting morphogenesis *in vitro*. Here, we show for the first time that meningioma cells support endothelial sprouting even under reduced exogenous growth factor conditions, suggesting that tumor-derived factors are sufficient to promote angiogenic behavior in this setting.

Merlin loss leads to constitutive mTORC1 hyperactivation,^13^ and we previously demonstrated that the third-generation, bi-steric inhibitor RMC-6272 achieved more specific and potent suppression of the mTORC1 signaling pathway compared to first-generation rapalogs.^17^ In this study, we demonstrate that mTORC1 inhibition by RMC-6272 results in a profound reduction in endothelial sprouting and network complexity in our 3D tumoroid model as well as significant downregulation of *APLN* mRNA expression. In addition, we show that exogenous Apelin stimulation enhanced sprouting in our 3D tumoroids, which supports its potential role in tumor angiogenesis in this system. This was further supported by a CRISPR-mediated *APLN* knockout model, in which *APLN*-null tumoroids exhibited a marked reduction in endothelial sprouting and network formation. Notably, prior studies have demonstrated Apelin-driven neo-angiogenesis, vascular remodeling, and resistance to anti-angiogenic therapy,^42^^,^^44-46^ thus raising the possibility that this pathway may contribute to disease progression and/or therapeutic resistance in meningioma as well.

mTORC1 is known to govern pro-angiogenic factors, including *IGF2*^48^  *and VEGFC*,^49^ which we observed to be increased in our transcriptomic analysis (Table 1). In the context of *NF2*-deficient meningioma, constitutive mTORC1 activation may thus promote a pro-angiogenic state, sustaining a milieu of angiogenic drivers beyond *VEGFA*, including Apelin. Consequently, the potent anti-angiogenic activity of RMC-6272 observed in our study likely results from the simultaneous suppression of this coordinated signaling network. Although additional studies will be required to further define the wider spectrum of changes, overall, our findings showing significant attenuation of endothelial sprouting in our tumoroid model, either by treatment with RMC-6272 or CRISPR knockout of *APLN*, position Apelin as a functionally relevant downstream component of mTORC1-driven angiogenesis in *NF2*-deficient meningiomas. It will also be interesting to examine the role of these angiogenic pathways in *NF2*-expressing meningioma in future studies employing our 3D tumoroid system.

In conclusion, we have developed a novel *NF2*-deficient 3D tumoroid co-culture model, which represents a robust and tractable platform for mechanistic interrogation. Employing this model, we have identified significant endothelial sprouting, which was disrupted upon mTORC1 inhibition or knockdown of Apelin. It will be of interest in future studies to examine the contribution of endothelial cells vs tumor cells toward the endothelial sprouting phenotype to potentially define the mechanism of mTORC1 inhibition that we observe. Given that mTORC1 signaling has been implicated more broadly in tumor angiogenesis, along with our data revealing mTORC1 as a potential driver of Apelin expression, our study supports a rationale to therapeutically target mTORC1-regulated angiogenic pathways in NF2-related meningiomas.

## Supplementary Material

vdag218_Supplementary_Data

## Data Availability

Relevant data will be made available upon request.
