# Supplementary material for: A 3D in vitro co-culture model to investigate tumor-endothelial interactions in Neurofibromatosis type 2-associated meningiomas
Source: Neurooncol Adv. 2026 Aug 17;8(1):vdag218. doi: 10.1093/noajnl/vdag218 (PMC13568822; doi:10.1093/noajnl/vdag218)
Supplement: Supplementary file 1 [file vdag218_supplementary_data.zip › Supplementary file_highlighted copy.docx]

**Supplementary Fig. 1.** Representative image of a vascularized MN1-LF-HUVEC tumoroid cultured in basal media without exogenous growth factors, showing endothelial sprouting from the spheroid core indicating meningioma cell-derived signals are sufficient to sustain angiogenic sprouting in the absence of exogenous angiogenic supplementation.

**Supplementary Fig. 2.** *APLN* expression in publicly available meningioma cohort stratified by recurrence status and WHO grade. (A) *APLN* mRNA expression in recurrent and non-recurrent meningiomas from the Nassiri et al. cohort (n = 121). *APLN* expression was higher in recurrent tumors compared with non-recurrent tumors (median expression: 1.24 vs. 0.34; P = 0.019). (B) *APLN* mRNA expression across WHO grade 1, 2, and 3 meningiomas. APLN expression demonstrated a progressive increase across WHO grades (median expression: 0.73, 1.16, and 1.24, respectively), although this trend did not reach statistical significance. Data is visualized using cBioPortal. Each dot represents a meningioma.

**Supplementary Fig 3.** Representative immunoblot demonstrating APLNR expression across the meningioma cell lines tested, including NF2-intact arachnoid cells (ACs), immortalized meningioma lines Ben-Men-1, MN1-LF, and KT21-MG, and primary meningioma lines MN597, MN619, MN621, MN626, and MN647D. β-actin was used as a loading control.

**Supplementary Fig. 4.** Representative fluorescent IHC images of a grade I meningioma section showing APLNR (green), nuclear counterstaining with DAPI (blue), and co-staining with the endothelial marker CD31/PECAM (magenta). The merged image demonstrates the spatial association of APLNR with CD31-positive vascular structures.

**Supplementary Fig 5.** Bar graph demonstrating the effect of Apelin-13 on number of junctions showing an upward trend upon stimulation with Apelin-13. All data are normalized to unstimulated control.

**Supplementary Fig 6.** Representative Sanger sequencing chromatograms showing the targeted *APLN* locus in wild-type and edited Ben-Men-1 clones. The null clone contains a single-nucleotide insertion (“T”) in exon 1, predicted to generate a frameshift and premature stop codon consistent with loss of function.
